# Effects of memory training or task design? A Commentary on “Neural evidence for the use of digit-image mnemonic in a superior memorist: an fMRI study”

**DOI:** 10.3389/fnhum.2015.00183

**Published:** 2015-04-01

**Authors:** Natasha Sigala

**Affiliations:** Department of Psychiatry, Brighton and Sussex Medical School, and Sackler Centre for Consciousness Science, University of SussexBrighton, UK

**Keywords:** expertise, memory, frontopolar cortex, fMRI, number processing

Mathematical experts generally fall in two broad categories: they may be individuals gifted with specific skills, but with deficits in social or other intellectual domains (savants) (Treffert, 2009), or people with mathematical and memory abilities within the normal range, who devote significant amounts of time and energy in practicing specific problems (e.g., memorizing digit sequences, practicing calendrical calculations). Importantly, they can be highly idiosyncratic in their problem solving approaches, and their exceptional abilities in maths do not generalize in other cognitive domains.

Yin et al. in this issue offer a rare insight of a gifted memorist (CL), who holds the Guinness World Record for reciting 67,890 digits of π since 2005 (Yin et al., 2015). Building on behavioral data from CL collected in 2006, the current study presents imaging data that shed light on the effects of the memory training on CL's brain networks several years after his intense preparation for breaking the record.

CL is a strategy memorizer who encodes two-digit groups as images (fixed associations for numbers 0–99, based on number shape or phonemic encoding, see Table 1A in Hu and Ericsson, 2012), and creates vivid stories with associations that connect them to each other and/or to physical locations (Hu and Ericsson, 2012). In spite of his exceptional memory for digits in π, he has average visual digit span memory (Hu et al., 2009). Crucially, the behavioral tasks employed by Yin et al. were designed to keep CL's performance similar to that of control participants, and do not tap into his ability for serial information memorization.

Although CL's accuracy performance in memorizing six two-digit numbers (study phase), and then judging which of two appeared earlier (recall phase) was indistinguishable from that of the control group, fMRI data revealed neural differences during study and recall for CL relative to controls. Specifically, during the encoding phase, bilateral frontal poles, left superior parietal lobule, left premotor cortex, insula, and left dorsolateral prefrontal cortex were more active compared to controls, while left middle and inferior frontal gyrus were less active. During the recall phase, there was higher activity in frontal, parietal and visual areas for CL relative to controls.

The authors highlight the finding of frontal pole engagement in CL in the two-digit study and recall conditions and interpret it in terms of retrieval of the practiced digit-image associations. However, based on results from single unit and lesion experiments, one would not expect the pair associates to be represented in prefrontal (Rainer et al., 1999; Sigala et al., 2008), but rather in temporal cortex areas (Miyashita, 1988; Takeda et al., 2005). Prefrontal areas though are important for top-down modulation of posterior sensory and memory areas (Hasegawa et al., 1998; Hon et al., 2009). Crucially, the frontopolar (FP) engagement in the two-digit task was also found in control participants during the recall phase. Perhaps then the FP activation is not best explained by the digit-image mnemonic training, but by the fact that the task required holding the study phase information in working memory and making self-generated decisions in the recall phase, in agreement with FP cell activity patterns reported by Tsujimoto et al. (2010). Unlike neurons in other prefrontal areas, FP cells specifically encode goals that require synthesis of sensory instruction and cognitive processing, and monitor the current status of each goal, e.g., as completed or impending (Tsujimoto et al., 2011). The participants in Yin et al. not only needed to maintain the serial order of the two-digits presented, but also had to judge which digits appeared earlier twice in the recall phase. This required them to keep the digit order and two judgment goals in mind at the same time, before they had to study a new array of six two-digit numbers a few seconds later.

Overall, given the rarity of the data from such unique individuals and their quirky approaches to problem solving, it is useful to have neural evidence that intense training changes brain network recruitment even in the context of different tasks and is evident several years after the training intensity has eased. It would be ideal to complement the univariate analysis with a multivariate approach (e.g., Minati and Sigala, 2013). In this recent study of an expert calculator, a multivariate analysis (graph-based mapping of effective connectivity), but not the univariate analysis, showed distinct cortical hubs in occipital and temporal areas involved in processing well-practiced problems. In contrast, hubs in frontal areas (prefrontal, orbitofrontal, and anterior cingulate) were associated with processing less-practiced problems. This combination overcomes the limitations inherent in the localization approaches, and offers insights in the network architecture and neural context within these cognitive processes take place, as well as in the problem solving strategies used by experts.

## Conflict of interest statement

The author declares that the research was conducted in the absence of any commercial or financial relationships that could be construed as a potential conflict of interest.
